# miR-29c contribute to glioma cells temozolomide sensitivity by targeting O^6^-methylguanine-DNA methyltransferases indirectly

**DOI:** 10.18632/oncotarget.10357

**Published:** 2016-07-01

**Authors:** Songhua Xiao, Zhen Yang, Xingsheng Qiu, Ruiyan Lv, Jun Liu, Ming Wu, Yiwei Liao, Qing Liu

**Affiliations:** ^1^ Department of Neurology, Sun Yat-Sen Memorial Hospital, Sun Yat-Sen University, Guanzhou, Guangdong, China; ^2^ Department of Hypertension and Vascular Disease, The First Affiliated Hospital, Sun Yat-Sen University, Guangzhou, Guangdong, China; ^3^ Department of Radiation Oncology, Sun Yat-Sen Memorial Hospital, Sun Yat-sen University, Guangzhou, Guangdong, China; ^4^ Department of Neurosurgery, Xiangya Hospital, Central South University, Changsha, Hunan, China

**Keywords:** glioblastoma, TMZ, MGMT, miR-29c, chemoresistance

## Abstract

Temozolomide (TMZ) is the most commonly used alkylating agent in glioma chemotherapy. However growing resistance to TMZ remains a major challenge to clinicians. The DNA repair protein O^6^-methylguanine-DNA methytransferase (MGMT) plays critical roles in TMZ resistance. Promoter methylation can inhibit MGMT expression and increase chemosensitivity. Here, we described a novel mechanism regulating MGMT expression. We showed that miR-29c suppressed MGMT expression indirectly via targeting specificity protein 1 (Sp1). MiR-29c overexpression increased TMZ efficacy in cultured glioma cells and in mouse xenograft models. The miR-29c levels were positively correlated with patient outcomes. Our data suggest miR-29c may be potential therapeutic targets for glioma treatment.

## INTRODUCTION

Glioblastoma multiforme (GBM) is the most common and aggressive type of brain malignancies, which characterized by relapse and resistance even with the combination of radio- and chemotherapy. It remains mostly incurable and has about 1 year median survival post-diagnosis [1, 2]. To data, first-line therapy for GBM comprises surgery with the maximum feasible resection, followed by radiotherapy and chemotherapy with alkylating agents such as temozolomide (TMZ) [3-5]. TMZ have shown the most activity against malignant glioma. Because TMZ readily cross the blood-brain barrier, so it exhibits most activity against malignant glioma and fewer adverse effects compared with other drugs.

However, TMZ is not always effective because of intrinsic or acquired chemoresistance of GBM cells. Identifying the diverse mechanisms of chemoresistance operating in human tumors and developing effective strategies to overcome resistance in our studies. TMZ is a most widely used alkylating agent that modifies DNA in several positions, one of them being O6-methylguanine MeG (O6MeG). This modified guanine preferentially pairs with thymine during DNA replication that initiate the DNA mismatch repair (MMR) pathway, which ultimately causes DNA double-strand breaks and induction of apoptosis [6-8]. The methylation damage induced by these agents can be reversed by O6 methylguanine-DNA methyltransferase (MGMT) [8]. MGMT is a suicide cellular DNA repair protein that rapidly reverses alkylation at the O6 position of guanine, transferring the alkyl group to a specific cysteine residue in the protein's active site [9, 10]. In this form, the enzyme is inactive in normal tissues, but high levels of MGMT activity are associated with resistance to alkylating agents in tumors [11]. A growing number of studies implicate MGMT removes cytotoxic O6MeG lesions induced by TMZ so that responsible for chemoresistance to TMZ in GMB cells [12-14]. High MGMT expression and abnormal MMR function are mechanistically linked to TMZ resistance in multiple tumor models, and elevated MGMT expression or lack of MGMT promoter hypermethylation in patient tumor specimens is associated with a worse outcome in patients with GBM treated with TMZ. Many studies have shown that a deficiency of MGMT can increase the sensitivity of high grade glioma to alkylating agents [15-18].

MicroRNAs (miRNA or miR) are an evolutionarily group of endogenous small noncoding RNAs that are involved in the post-transcriptional regulation of gene expression by targeting mRNAs [19-21]. MiRNAs play crucial roles in regulating most biological processes of normal development and various diseases, including cancers, by suppressing mRNA stability and/or translation [22, 23]. Growing evidences support that miRNAs are aberrant expressed in human cancer tissues. MiRNAs also regulate DNA damage response and DNA repair, interfering with the response to chemotherapy or radiotherapy [24]. Several studies have implied that the modulation of miR expression levels is a feasible therapeutic strategy for cancer [25-27].

One particular miRNA family, the miRNA-29 family suppress DNA methylation of tumor-suppressor genes [28-30], reduce proliferation of tumors and increase chemosensitivity. Previous research has shown that the family member miR-29b functions as an oncogene by inhibiting tumor suppressors such as phosphatase and tensin homolog (PTEN) [30]. In this report, we provide evidence that miR-29c regulate MGMT expression indirectly via targeting of transcription factor Specificity Protein 1 (Sp1), a universal zinc finger transcription factor that in expressed in nearly all cells and tissues [31, 32]. We show that the SP1 expression increasing the response to TMZ in glioma cell lines. We studied the relationship between the MGMT expression and the resistance to TMZ, our findings may help to design new drugs or new therapeutic regimen to overcome chemo-resistance to TMZ.

## RESULTS

### miR-29 family is downregulated in primary glioma tissues

**Figure 1:**
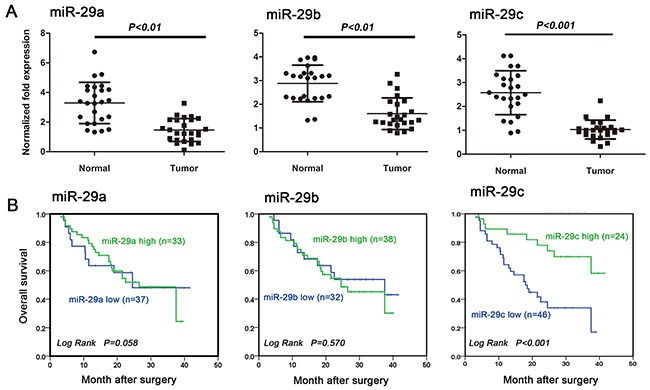
MiR-29c is an independent prognostic factor for glioma patient overcome **A.** miR-29a, miR-29b and miR-29c expression were downregulated in human glioma tissues examined by using qRT-PCR. Tumor: glioma tissues; Normal: the matched normal brain tissues. **B.** Kaplan-Meier survival curves for OS in relation to miR-29a, miR-29b and miR-29c expression. Cutoff values for miR-29s (high/low expression) were determined by ROC analysis by using SPSS16.0 software.

To explore the roles of miR-29 family in glioma pathogenesis, we first quantified miR-29 levels in 22 primary glioma tissue samples. We found that miR-29a, miR-29b, and miR-29c were significantly downregulated in the majority of tumor samples when compared with the matched adjacent normal brain tissues (Figure 1A).

### miR-29c expression is associated with patient survival and tumor recurrence

In order to study the relationship between miR-29c expression and patient prognosis, we performed in situ hybridization on a cohort of 70 defined human glioma samples since the survival information is not available for the above 22 cases. We observed that higher miR-29c level was associated with better overall survival (OS) (Figure 1B; P<0.001). No significant correlation was observed between miR-29a (Figure 1B; P=0.863) or miR-29b level and the outcome (Figure 1B; P=0.570). We further examined whether TMZ chemotherapy would be more beneficial in patients with high miR-29 expression. We followed 48 patients who underwent TMZ treatment after maximal tumor resection. 25 of them had recurrent tumors. We found that patients with grade III or IV gliomas had low miR-29 levels (miR-29a: P= 0.017; miR-29b: P= 0.016; miR-29c: P= 0.001). However only miR-29c level was strongly (inversely) associated with tumor recurrence (P=0.001), and no such relationship was observed for miR-29a (P=0.784) or miR-29b (P=0.774) (Table 1). There is no gender difference in miR-29 expression.

**Table 1:** Analysis of the correlation between expression of miR-29a, miR-29b and miR-29c in primary glioma and its clinicopathological parameters

	Cases	miR-29a			miR-29b			miR-29c		
		low	high	P	low	high	P	low	high	P
TMZ response										
sensitive	23	15	13	0.784	11	12	0.774	7	16	0.001
resistance	25	11	14		16	9		23	2	
Gender										
Male	37	17	20	0.362	16	11	0.219	23	14	0.457
Female	33	12	11		12	21		17	16	
Glioma histopathology										
Grade I-II	22	10	12	0.017	8	14	0.016	13	9	0.001
Grade III-IV	48	27	21		24	24		33	15	

### miR-29c increased TMZ sensitivity in vitro

**Figure 2:**
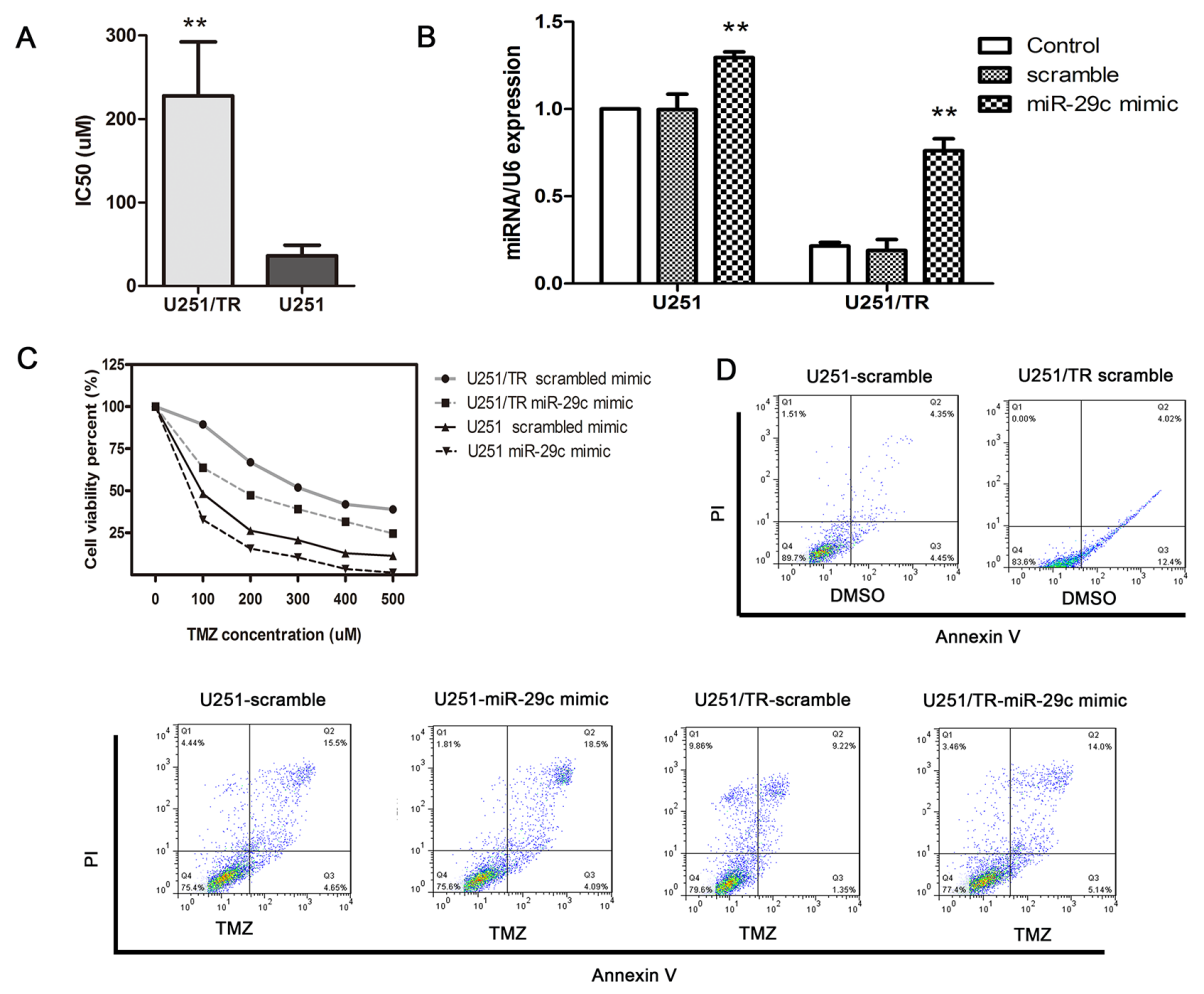
Ectopic expression of miR-29c increased TMZ sensitivity by inhibiting cell growth and promoting apoptosis in U251/TR cells **A.** U251/TR and U251 cells were exposed to various concentrations of TMZ for 48 hours, and the 50% inhibitory concentration (IC50) of TMZ was examined by the MTT assay. **, *P* < 0.01. **B.** U251/TR and U251 cells were transfected with miR-29c mimics or scramble, miR-29c expression level was assessed by qRT-PCR. **, *P* < 0.01. **C.** At 48 hours after transfection with miR-29c mimics or a scramble control, cells were exposed to a range of TMZ concentrations for another 48 hours, after which cell viability was determined with an MTT assay. **D.** The scramble-transfected and miR-29c-transfected cells were treated with TMZ or DMSO, after 48 h of the treatment, cells apoptosis was evaluated using flow cytometry. All results are expressed as the mean ± SD of three independent experiments.

We further investigated the effect of miR-29c on TMZ sensitivity. We first compared viability of U251 cells (TMZ sensitive) with that of U251/TR cells (TMZ resistant) using MTT assay. The IC50 is 6.4 fold higher in U251/TR cells than U251 cells after 10-day TMZ exposure (Figure 2A). Then we quantified miR-29c levels in both cell lines. We found its expression was significantly decreased in U251/TR cells (Figure 2B). It indicated that low miR-29c expression in U251/TR cells was associated with the chemo-resistance. We then transfected both cell lines with miR-29c mimics or scramble control before the 48-hour exposure of TMZ at various concentrations. We noticed that IC50 of U251/TR cells was significantly decreased in the experimental group compared with the control transfection group (Figure 2C). miR-29c transfection even made U251 cells more sensitivity to TMZ. The flow cytometry assay showed that there were significantly more apoptotic cells in the experimental group (Figure 2D). These data suggested that miR-29c overexpression could increase TMZ sensitivity by inducing apoptosis.

### miR-29c increases TMZ sensitivity in in vivo

**Figure 3:**
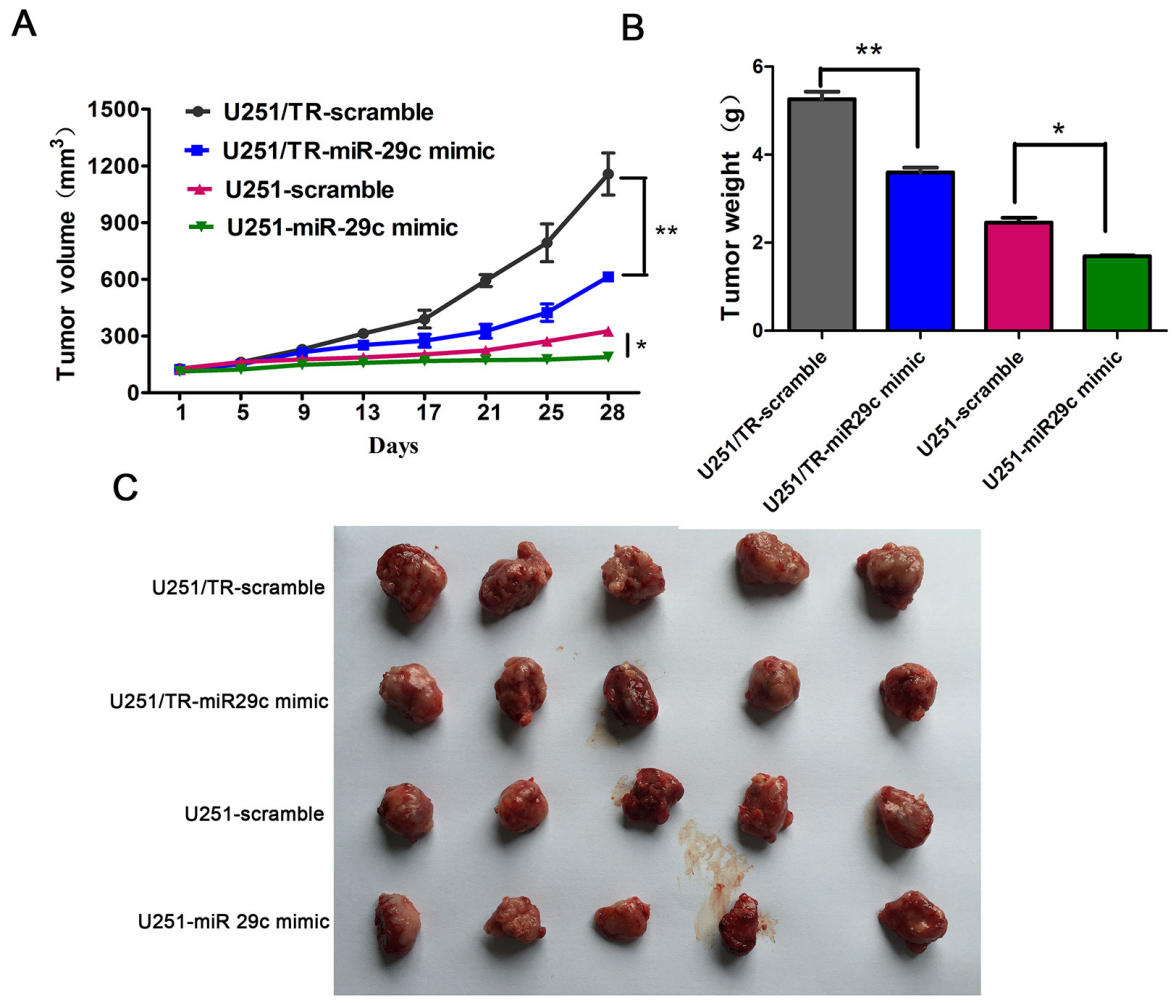
The overexpression of miR-29c enhanced TMZ sensitivity in vivo **A.** Size of subcutaneous tumor growth after injection of scramble-transfected and miR-29c-transfected resistant U251/TR cells and sensitive U251 cells, then followed by TMZ therapy or DMSO for 4 weeks. Tumor size was assessed every four days. *, *P* < 0.05, **, *P* < 0.01. **B-C.** After four consecutive weeks of therapy, the mice were euthanized and the wet weights of the tumors were recorded. Representative images of xenografts (C) and a summary of tumor weight in nude mice (B). *, *P* < 0.05, **, *P* < 0.01. All results are expressed as the mean ± SD of three independent experiments.

To investigate the effect if miR-29c in vivo, U251 or U251/TR cells with or without miR-29c transfection were injected subcutaneously into the nude mice. The tumor size was similar among different mice 3 weeks after injection which showed minimal variation between injections. Then TMZ was administered twice a week at a dose of 10mg/kg. After 4 consecutive weeks of treatment, the mice were euthanized. The tumors were excised, and the wet weights of the tumors were recorded (Figure 3A-3C). The tumor size or weight was significantly smaller or lighter in mice injected with miR-29 transfected glioma cells. These data suggested that miR-29c can increase TMZ sensitivity in vivo.

### miR-29c indirectly targeting MGMT through Sp1

**Figure 4:**
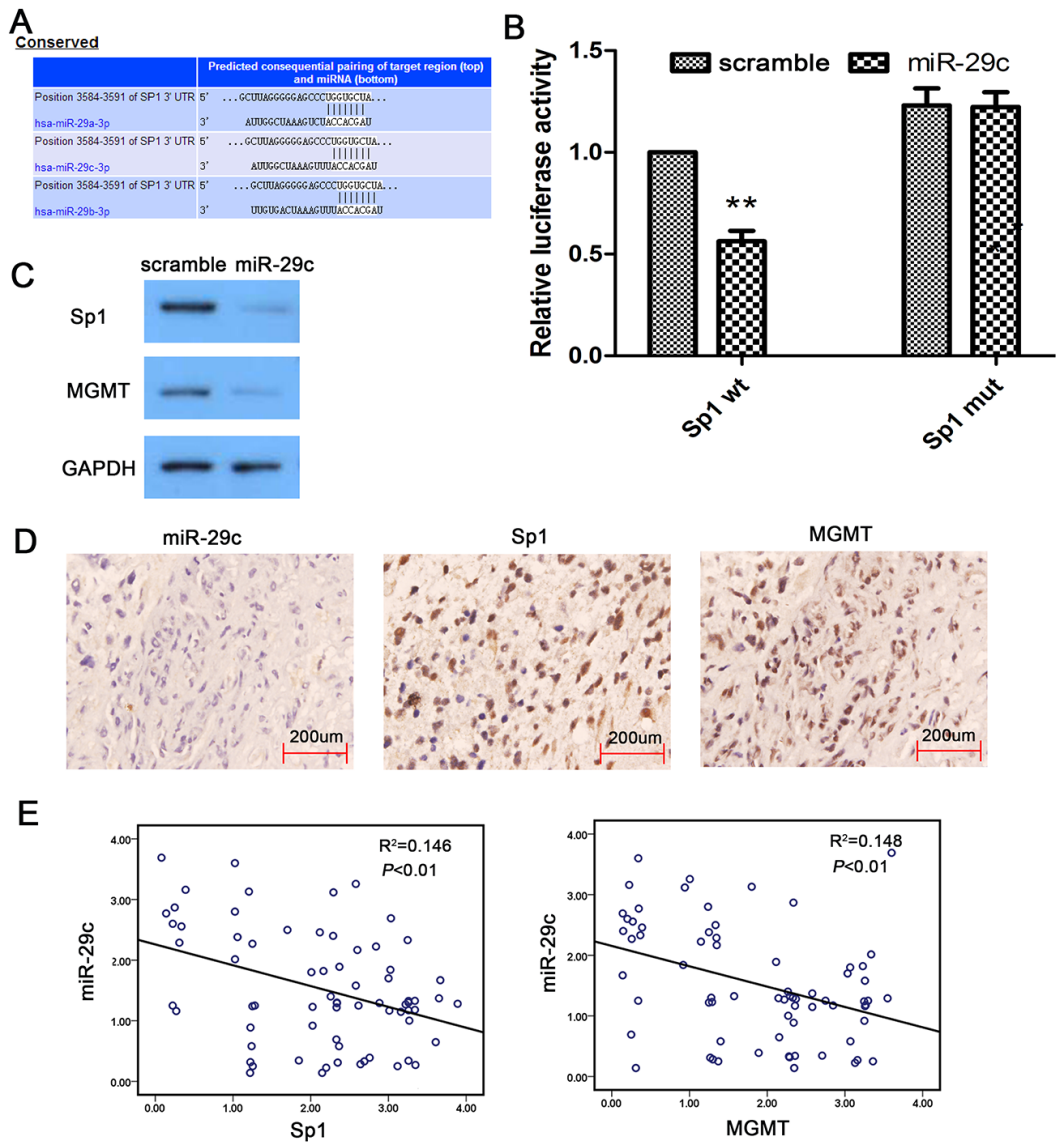
MiR-29c indirectly target MGMT in human glioma **A.** The base-pairing interaction of miR-29c seed sequences and Sp1 as predicted by bioinformatics analysis. **B.** MiR-29c inhibit wild-type (wt) but not mutated (mut) Sp1-3′-UTR reporter activity. An empty luciferase reporter construct was used as a negative control. *, *P*<0.05. **C.** U251/TR cells was transfected by scramble, miR-29c mimic for 24 hours. Expression of Sp1 and MGMT in cells were determined using Western blot assay (normalized to GAPDH). **D.** Human glioma specimens were analyzed by ISH and immunohistochemical staining, and the representative miR-29c, Sp1 and MGMT expression is shown. **E.** Analysis of immunohistochemistry data showing linear regressions and inversely correlations of miR-29c with Sp1 and MGMT in human glioma tissues.

To understand how miR-29c increases TMZ sensitivity in glioma cells, we conducted an RNA hybrid alignment bioinformatics search and predicted a binding site for miR-29c at the position 3584-3591 of the 3′-UTR of Sp1 (Figure 4A). We then performed luciferase reporter assay to confirm our prediction. We cloned the wild-type or the mutated 3′-UTR of Sp1 into the luciferase reporter vector. We found that miR-29 mimic transfection remarkably suppressed luciferase activity in the vector containing wild-type Sp1 sequence. This was not observed in the vector containing mutated Sp1 sequence (Figure 4B). The data suggested that miR-29c directly bound to Sp1 DNA. We also found that the protein levels of Sp1 and MGMT was significantly reduced in U251/TR cells after miR-29c mimic transfection (Figure 4C). Previous studies showed that Sp1 upregulated MGMT expression by increasing MGMT promoter activity. Our results indicated that miR-29c indirectly suppressed MGMT expression by targeting Sp1 in glioma cells. Using Spearman correlation analysis, we examined the association between endogenous miR-29c and Sp1, MGMT immunostaining intensity in human glioma tissues (Figure 4D). We discovered an inverse relationship between miR-29c and Sp1/MGMT levels in tumor samples (Figure 4E). Our data further supported a mechanistic link between miR-29c-mediated Sp1 downregulation and the subsequent decrease in MGMT expression.

## DISCUSSION

While multiple mechanisms that mediate intrinsic or acquired resistance to TMZ have been recognized, MGMT is now been recognized as a major role in mediating TMZ and other alkylating agent resistance [33]. Plenty of evidence suggests that the intracellular level of the alkylating enzyme MGMT interfere TMZ response in GMB patients [11, 34]. Low levels of MGMT expression are associated with a better TMZ response, because without the MGMT, cells are not able to repair the TMZ-induced base mismatch. Consequently, DNA mismatch repair, double-stand DNA breaks, and the apoptotic pathway are activated. MGMT expression is regulated by the methylation of its promoter, and low promoter methylation accounts for a greater TMZ response when associated with radiotherapy in GMB patients. Therefore, MGMT promoter methylation may predict TMZ resistance. Overcoming TMZ resistance is an urgently expected development in cancer therapeutic, several studies are also investigating avenues to overcome TMZ resistance by regulation MGMT promoter methylation or gene expression level.

In the present study, we addressed this specific issue by investigating the involvement of miRs in MGMT regulation. We found that miR-29c mediated MGMT repression could be mediated via Sp1. The results also suggested that miR-29c can enhance resistance to TMZ chemotherapy in glioma cells. Several human miRs, including miR-29 family, miR-148, and miR-200b/c have been found to be frequently downregulated in human cancers and lead to increase expression of DNMT1 and DNMT3a/b because they directly target the 3′-UTR of DNMTs [30, 35, 36]. The human miRNA-29 family has three main members including miR-29a, miR-29b and miR-29c. Transcriptional profiling studies of miRNA expression across tumor tissues or cancer cell lines have revealed that miR-29 is downregulated in the majority solid tumors. In the nervous system, miR-29b was found to be downregulated in glioblastomas [37] and neuroblastoma [38]. MiR-29 has been shown to induce apoptosis and inhibit proliferation and invasion by downregulating oncogenes and/or upregulate tumor suppressors. One of commonly acknowledged anti-oncogenes mechanism is demethylation of oncogenes and methylation. The miR-29 family has a finely complementary structure to the 3′-TURs of DNA methyltransferase DNMT3a and DNMT3b [39]. Garzon et al found that miR-29b not only directly bound to DNMT3a/b but also indirectly suppressed DNMT1 by binding to Sp1. Sp1 is a transactivator of the DNMT1 gene. Moreover, Sp1 can also access the MGMT promoter and set the transcriptional state of the MGMT gene [40]. Plenty of evidence showed that Sp1 overexpression in tumor relieved P53 mediated down-regulation of MGMT, the fact is P53 competes with SP1 for binding to the same sequence present on the MGMT promoter [41]. This suggests that sequestration of Sp1 could be one of the avenues to enhance sensitivity of tumor cells to alkylating generating drugs.

In summary, we have provided evidence of the existence of an adjunct mechanism of MGMT regulation, besides promoter methylation, involving miR targeting oncogenes. This supports the conception that promoter methylation is not the only regulatory mechanism of MGMT expression [42, 43]. We also showed that overexpression of miR-29c produces an increase in sensitivity to TMZ via a reduction in the level of MGMT. On the other hand, these studies provide a possible mechanism of miR-29c may potentially serve as a novel and effective therapeutic agent for overcoming TMZ resistance in glioma patients.

## MATERIALS AND METHODS

### Pathological samples and cell lines

Tumor samples and the corresponding adjacent tissues harvested from 21 glioma patients were stored at −80°C before RNA extraction. Experimental procedures of human tissue were conducted in compliance with the declaration of Helsinki. The research was approved by the institutional review boards (IRBs) of Central South University. Informed consent was obtained from patients after explanation of the research study. Tissues for ISH and IHC were collected as previously described [44]. Human glioblastoma-derived cell lines U251 was obtained from the Cell Research Institute of Peking Union Medical College (Peking, China) and maintained in Dubecco's modified Eagle's medium/high glucose (Invirogen, Carlsbad, CA) supplemented with 10% fetal calf serum (GIBCO), 1% penicillin and streptomycin (Life Technologies). The U251-TMZ resistant cell line (U251/TR) was cultured and evaluated the resistance factor as previously [45]. Cells treated with DMSO served as controls. All cells were cultured in a 37°C humidified incubator supplied with 5% CO_2_.

### Transfections and dual luciferase assay

Cells were seeded in six-well plates at 1×10^5^ cell/well followed by cultured for 24 hours and transfected with 20nmol/L miR-29c mimic or negative control mimics (NC), using lipofectamine 2000 (invitrogen). The NC was synthetic scrambled double oligonucleotides, non-targeting against to any mRNA. The effect of mimics was examined in triplicate at 24h post-transfection. The dual luciferase reporter assay (Promega Corp.) was performed 48 hours after transfection according to the manufacturer's protocol.

### Cell viability assay

MTT assay was used to determine the cell viability and the IC_50_ of TMZ in both parental U251 cell line and U251/TR cell line according to the manufacturer's instructions. The assay was conducted as follows: non-transfected or transfected cells were re-seed into 96-well plates, 24 hours later, freshly prepared TMZ (Sigma Chemicals, St Louis, MO, USA) at various concentrations was added and cells were cultured for an additional 48 hours. Optical density was measured at the wavelength of 540 nm on a microplate reader (Bio-Rad Laboratories). At least three independent experiments were performed in quadruplicate.

### In situ hybridization (ISH) and immunohistochemistry (IHC)

The miR-29a, miR-29b and miR-29c miRCURYTM LNA custom detection probe (Exiqon, Vedbaek, Denmark) was used for ISH. ISH procedures were performed as previously described. Paraffin blocks of tumors were sectioned into 5 um slices and then processed using standard techniques and the IHC process was described as previously [35]. The staining intensity was scored as follows: 0-1 (no staining), 1-2 (weak staining), 2-3 (medium staining) and 3-4 (strong staining). The percentage of positive cells was divided into four rankings: 0%-25%; 26%-50%; 51%-75%; and 76%-100%. The expression score was calculated by multiplying the intensity score with the percentage. All specimens were independently evaluated by at least two pathologists in a double-blind fashion. Expression scores equal to or greater than 2 was considered as high expression, and smaller than 2 as low expression.

### Quantitative real-time PCR

Total RNA was extracted from the cells with the Trizol Reagent (Invitrogen, Carlsbad, CA, USA). Quantitative real-time reverse-transcription polymerase chain reaction (qRT-PCR) was performed using the All-in-One™ miRNA qRT-PCR detection kit (GeneCopoeia, Rockville, MD, USA) for miR-29 and small nuclear RNA U6, which was used as an endogenous control. The PCR cycle parameters were as follows: 95 °C for 15 min, 39 cycles of denaturation at 95 °C for 15s, annealing at 50 °C for 30s, and extension at 70 °C for 30s. For mRNA expression analysis, cDNA was synthesized using cDNA reverse transcription kit (Thermo Fisher Scientific, MA, USA) and a PCR analysis was performed using QuantiFast SYBR Green PCR Kit following the manufacturer's instructions. The PCR cycle parameters were as follows: denaturation at 95 °C for 5 min, 39 cycles of denaturation at 95 °C for 10s, annealing at 60 °C for 30s, and extension at 72 °C for 30s. The real-time PCR reactions were performed in triplicate. The specific primers for miR-29a (Catalog# HmiRQP0371, Genecopoeia, USA), miR-29b (Catalog# HmiRQP0373, Genecopoeia, USA) and miR-29c (Catalog# HmiRQP0375, Genecopoeia, USA) were purchased from GeneCopoeia. The relative expression levels were calculated using the 2^−ΔΔct^ method.

### Western blot

The cells were lysed in RIPA buffer in the presence of proteinase inhibitor on ice (Sigma-Aldrich, St. Louis, MO, USA). Lysates were centrifuged and SDS gel loading buffer was added. Equal amounts (20mg) of protein was separated by 12% SDS-PAGE and transferred to PVDF membrane (Millipore Corp, Billerica, MA, USA). Membranes were blocked with 10% skimmed milk followed by incubation with the primary antibodies overnight at 4°C. The antibodies against Sp1 and MGMT (1:500 dilution) purchased from Santa Cruz Biotechnology. An anti-GAPDH antibody (MAB; Millipore, CA, USA) was used as a protein loading control. After washed extensively with 0.1% PBS, the membranes were incubated with secondary antibodies.

### Flow cytometry-based apoptosis

Cells were cultured in the medium added with TMZ and incubated for 48 hours. After incubation, the cells were harvested, stained with annexin V-fluorescein isothiocyanate (FITC) and propidium iodide (PI). The mixture was incubated at room temperature in the dark for 15 min and analyzed by FACS.

### Nude mice model

Approximately 10^7^ cells were injected intraperitoneally into nude mice. One week later, TMZ therapy was initiated at a dose of 10 mg/kg twice per week. Values for the tumor volume (V) were determined by measuring the longitudinal cross section (L) and the transverse section (W) and then applying the formula V = (L × W^2^)/2. After the initial treatment, the tumor size was determined every day. After four consecutive weeks of therapy, the mice were euthanized and the wet weights of the tumors were recorded.

### Luciferase reporter assay

The full length 3′-UTR of Sp1 was amplified by PCR from genomic DNA and inserted into the pGL3 control vector (Pro-mega) using the XBA1 site immediately after the stop codon of luciferase. The potential miR-29 binding site in the 3′-UTR of Sp1 was mutated by the overlap extension PCR method. Primer sequences are: F 5′-CCTTCAGGGATTTCCAACTG-3′ and R 5′ GTCCAAAAGGCATCAGGGTA-3′**.** Both wild type and mutant 3′-UTRs were ligated into PsiCheck2 plasmid (Promega) at Xhol and Not1 sites directly downstream the luciferase coding sequence. Cells were cotransfected with 500 ng of PGL3-Sp1-WT or PGL3-Sp1-Mut constructs with miR-29c mimics or a scramble control. Each sample was cotransfected with pRL-TK plasmid to monitor the transfection efficiency. Luciferase activity was examined 48 hours after transfection with the dual luciferase reporter assay system (Promega).

### Statistical analysis

All statistical analyses were performed using SPSS 17.0 (SPSS Inc, Chicago, IL). The pearson χ2 test was used to study relationships between variables, such as miR-29c expression levels in tumor samples. Differences between samples were analyzed using the two-tailed student's t-test. The SPSS software was also used to calculate the IC50 based on the dose-response curve. Statistical significance was accepted at P<0.05.
